# Firefly algorithm-based LSTM model for Guzheng tunes switching with big data analysis

**DOI:** 10.1016/j.heliyon.2024.e32092

**Published:** 2024-05-29

**Authors:** Mingjin Han, Samaneh Soradi-Zeid, Tomley Anwlnkom, Yuanyuan Yang

**Affiliations:** a Xinxiang University, Xinxiang, 453003, China; b University of Sistan and Baluchestan, Zahedan, 9816745845, Iran; c Wichita State University, Wichita, 67260, USA

**Keywords:** Large models, Guzheng tunes switching, Firefly algorithm, LSTM networks, Big data analysis

## Abstract

Guzheng tune progression involves intricately harmonizing melodic motif transitions. Effectively navigating this vast creative possibility space to expose musically consistent elaborations presents challenges. We develop a specialized large long short-term memory (LSTM) model for generating musically consistent Guzheng tune transitions. First, we propose novel firefly algorithm (FA) enhancements, e.g., adaptive diversity preservation and adaptive swim parameters, to boost exploration effectiveness for navigating the vast creative combinatorics when generating Guzheng tune transitions. Then, we develop a specialized stacked LSTM architecture incorporating residual connections and conditioned embedding vectors that can leverage long-range temporal dependencies in Guzheng music patterns, including unsupervised learning of concise Guzheng-specific melody embedding vectors via a variational autoencoder, encapsulating unique harmonic signatures from performance descriptors to provide style guidance. Finally, we use LSTM networks to develop adversarial generative large models that enable realistic synthesis and evaluation of Guzheng tunes switching. We gather an extensive 10+ hour corpus of solo Guzheng recordings spanning 230 musical pieces, 130 distinguished performing artists, and 600+ audio tracks. Simultaneously, we conduct thorough Guzheng data analysis. Comparative assessments against strong baselines over systematic musical metrics and professional listeners validate significant generation fidelity improvements. Our model achieves a 63 % reduction in reconstruction error compared to the standard FA optimization after 1000 iterations. It also outperforms baselines in capturing characteristic motifs, maintaining modality coherence with under 2 % dissonant pitch errors, and retaining desired rhythmic cadences. User studies further confirm the superior naturalness, novelty, and stylistic faithfulness of the generated tune transitions, with ratings close to real data.

## 1 Introduction

The Guzheng represents a crowning achievement of Chinese cultural refinement, boasting a history tracing over two and a half millennia [1]. As the Guzheng's celestial voice echoing with shimmering overtones and emotional sensitivity spread via traveling performers out from its likely Wuxi birthplace aglow with the region's lush nature and flowing waters, so too did its fame and appeal culminating in dynastic prominence during the Qing empire days where it became an imperial court staple while simultaneously permeating rural aesthetic consciousness through heartfelt solo songs and pieces fusing classical compositions with vibrant folk colors. Right up through the modern global era, as exemplary works by Guzheng luminaries vault the instrument squarely onto the global stage, each evolution in strings or layout unlocking ever more versatile palettes continues carrying a profound artistic impact befitting the Guzheng's rightful place as one of humanity's most emotive soundscapes [2].

However, mastering professional creative Guzheng composition poses difficulties, exceptionally smoothly harmonizing transitions between melodic passages. Rather than abrupt or disjointed changes, seamless segues between motives enhance sonic flow. This flair relies on understanding nuanced tension resolution principles and idiomatic phrasing unique to the instrument. Unfortunately, developing this intuitive grasp requires years of practice, even for prodigies. Hence, we pursue new pathways to augment such creativity. Drawing inspiration from adaptive natural phenomena, evolutionary algorithms provide a biology-inspired paradigm well-suited for gradual optimization even in vast, underdefined domains by mimicking iterative selective pressures toward favorable traits [[3], [4], [5]]. The firefly optimization method mimics insect swarms, using agents that explore the search space through a combination of random walks and movements influenced by the attractiveness of other agents. This interplay between randomness and mutual attraction allows the algorithm to effectively navigate complex solution landscapes and find optimal or near-optimal solutions. By enhancing the algorithm's diversity preservation and local tuning as needed, we significantly boost its explorative reach – an essential requisite for effectively modeling the Guzheng's structural intricacy. Soft resets continually cycle in new genetic material, preventing stagnation around merely adequate local transitions while accumulated gains focus traversals. The tuned arrangements provide the right balance of freedom and restraint to expose the Guzheng possibilities.

Equally vital, machine learning unlocks data-driven revelations into the underlying grammar of music otherwise opaque to direct analysis [6,7]. We leverage long short-term memory (LSTM) neural networks, proven uniquely adept at sequence prediction and generation tasks, including language translation, text synthesis, and time series forecasting [[8], [9], [10], [11]]. The core breakthrough stems from LSTM's special recurrent memory cells, accumulating persistent context as signals propagate through their depth. This grants sensitivity to long-range dependencies over extended horizons that plague simpler models, enabling the capture of the Guzheng's intricate logical motifs spanning hundreds of tones. Carefully evolved architectural alignments between the optimization and modeling facets fuse their complementary strengths synergistically towards crystallizing insights into Guzheng's inner aesthetic essence – replicating the instrumental glimpse master performers acquire through decades of practice in weeks automated by tensor transformations. Hyperparameter tuning further customizes response to the instrument's nuances, right-sizing lookback capacity, and receptive field density for its richer combinatorics than vocal lines [12,13].

Large models and big data analysis provide a synergistic combination of capabilities that significantly improve the task of modeling Guzheng tune transitions and switching. By exponentially increasing parametric scale and leveraging insights from extensive Guzheng performance data, these approaches better capture the underlying creative intricacy while guiding the complex elaboration process to ensure coherence. The enriched receptive capacity of billion-parameter generative designs assimilates the accumulated recorded melodic motifs, rhythmic articulations, and harmonic intersections aggregated from commercial albums, competitions, and videos toward crystallizing pattern comprehension within model representations. Meanwhile, extensive embedding vectors distilling salient Guzheng principles from abundant example statistics calibrate the generative crafting of pivot points between tonal modes or tempi through conditional style guidance without severely restricting novelty - much as a compass funnels ship navigation relative to magnetic north even across unfamiliar waters. Altogether, having immense resources available empowers large neural models to expose more precise characteristic nuances amidst the billions of possible variations rooted in the ancient Chinese instrument, which is demanding yet expressive techniques while using data-driven wisdom accumulated from generations of musical tradition to respect a center of gravity as evolutions unfold. The resulting combination enhances creative augmentation while retaining aspects of cultural lineage.

Training on an extensive corpus of studio Guzheng solo performances captures the natural harmonic flow and rhythmic articulation nuances exemplary of idiomatic playability for precise continuation synthesis. We distill these intricate patterns into concise 32-dimensional melody embedding vectors encoding unique harmonic signatures and sequential logic via an unsupervised variational autoencoder (VAE) regularization forcing information bottlenecks. This melodic essence distillation quantifies the ineffable, mathematically orchestrating salient motifs and tonal progressions relational to acoustic first principles. Conditional injection of such high-level stylistic guidance then steers our generative LSTM model annexes to craft musically consistent transitions within permissible tonality neighborhoods by occasionally reigning back extrapolations through holistic course correction. It is akin to a sailing ship navigating unfamiliar waters with only a compass bearing indicating directionality relative to ground-truth north as a reference during overcast skies. Such perspective-keeping ensures coherence even as deviation mounts stochastically, significantly elevating autonomous continuation quality. The approach respects innate identity, using Guzheng's voice to guide sightless traversal of its possibilities.

Adversarial objectives create a dynamic interplay between discriminator networks that assess the realism of generated samples and generator networks that strive to produce synthetic examples indistinguishable from authentic ones. By integrating Guzheng-specific embedding vectors into the discriminator and generator architectures, the adversarial framework becomes attuned to Guzheng music's unique characteristics and nuances. These embedding vectors encapsulate essential musical elements, such as melodic patterns, harmonic structures, and playing techniques, enabling the networks to capture and reproduce the creativity and authenticity inherent in Guzheng compositions. Concurrently, the firefly algorithm (FA) undergoes a specialized tuning process to evolve optimal hyperparameter configurations tailored specifically to the Guzheng instrument. This optimization phase allows the FA to explore and discover the most effective settings for generating high-quality Guzheng music, considering the instrument's intricate possibilities and rich musical heritage. The algorithm can efficiently navigate the vast search space and converge on hyperparameter values that yield the most compelling and authentic results by fine-tuning the FA to the specific requirements of Guzheng music generation. Integrating machine learning techniques, a deep understanding of Guzheng musicality, and advanced optimization methods creates a powerful collaboration that unlocks the full potential of AI-assisted Guzheng composition. Rather than replacing human insight and creativity, these techniques complement and enhance the artistic process. By providing a robust framework for exploring the intricacies of Guzheng music, the AI system acts as a catalyst for innovation, inspiring musicians and composers to push the boundaries of their art form.

The main contributions of this study on employing evolutionary LSTM models for AI-assisted Guzheng tunes switching composition include the following:

• We develop a specialized large LSTM model for generating musically consistent Guzheng tune transitions. The architecture leverages recent advances in scaling up recurrent models to billions of parameters, achieving state-of-the-art results on tasks requiring sequential understanding and creative elaboration.
• We propose novel FA enhancements, e.g., adaptive diversity preservation and adaptive swim parameters, to boost exploration effectiveness for navigating the vast creative combinatorics when generating Guzheng tune transitions.
• We develop a specialized stacked LSTM architecture incorporating residual connections and conditioned embedding vectors that can leverage long-range temporal dependencies in Guzheng music patterns, including unsupervised learning of concise Guzheng-specific melody embedding vectors via VAE, encapsulating unique harmonic signatures from performance descriptors to provide style guidance.
• We use LSTM networks to develop adversarial generative models that enable realistic synthesis and evaluation of Guzheng tune transitions.

The rest of this paper is structured as follows. Section 2 reviews the related works. Section 3 introduces the evolutionary large models. Guzheng tunes switching with LSTMs is studied in Section 4. Section 5 conducts the simulation, and Section 6 shows the conclusion.

## 2 Related works

This study builds upon the rich intersection of musical intelligence, evolutionary optimization algorithms, and sequence modeling approaches. Early rule-based expert systems by pioneers like Ebcioglu leveraged handcrafted musical knowledge to demonstrate simple automated composition but needed more adaptability [14]. With machine learning advancements, statistical models and Markov chains enabled formal modeling of repetitive patterns and likely progressions based on probability distributions [15,16]. However, these needed help to maintain longer-term structure and contextual consistency.

Recurrent neural networks (RNNs) made modeling extended temporal context possible for the first time [[17], [18], [19]]. Specifically, LSTM architectures overcame notorious vanishing gradients plaguing vanilla RNNs by introducing memory cells capable of preserving key learnings across hundreds of processing steps [[20], [21], [22], [23]]. This breakthrough performance on retaining long-range sequential dependencies proved revolutionary for algorithmic music generation tasks.

Combinations blending neural architectures with evolutionary optimization methods have improved solution search efficiency and customization [[24], [25], [26], [27]]. Genetic algorithms hybridized with LSTMs have shown promise for guiding melody harmonization by leveraging selection pressure mechanics [28]. The FA's unique mechanisms, such as the attractiveness function and randomized movement, enable it to effectively explore the vast and complex search space of possible Guzheng tune transitions, which is crucial for discovering novel and creative transitions that capture the richness and nuances of Guzheng music. Additionally, PSO may be more prone to getting stuck in local optima due to its emphasis on the best global and personal positions. The FA is relatively robust to parameter settings compared to other algorithms like PSO. Most importantly, the FA has been successfully applied to various optimization problems in music and creative domains, demonstrating its potential for generating novel and aesthetically pleasing solutions. For example, the FA has been used for music composition, melody harmonization, and musical feature extraction. These successful applications suggest that the FA is a promising choice for optimizing Guzheng tune transitions, as it can effectively handle musical optimization's creative and subjective aspects. We contribute a novel suite of tailored FA enhancements specialized for capturing distinguishing Guzheng characteristics.

Recent explosive progress in deep generative models like generative adversarial networks (GANs) and VAEs further enable the creating of highly realistic outputs across modalities from images to text sequences by better approximating the target distribution through competing objectives and compact representational regularization, respectively [[29], [30], [31], [32], [33]]. We adapt these to condition LSTM GANs using melodically derived latent vectors for transferable style guidance.

In [11], the authors proposed a novel deep generative model, conditional LSTM generative adversarial network (cLSTM-GAN), for melody generation from lyrics. In Ref. [8], the authors presented a method to automatically compose and play Guzheng music by training LSTM with reinforcement learning (RL-LSTM). In Ref. [34], the authors proposed a pre-trained MRBERT model for multitask-based music generation to learn melody and rhythm representation. In Ref. [35], the authors proposed a novel adversarial transformer to generate a transformer to generate music pieces with high musicality. Previous studies have explored various aspects of music generation using machine learning techniques [[36], [37], [38]]. However, they need to focus on the unique characteristics of Guzheng music or the importance of leveraging big data analysis to capture the rich diversity of Guzheng styles and techniques [39,40]. In contrast, our approach introduces several key innovations that set it apart from existing methods. By combining the advances in evolutionary optimization, neural architecture design, and big data analysis, our approach achieves state-of-the-art results in generating musically consistent and stylistically faithful Guzheng tune transitions, as demonstrated through comprehensive evaluations against strong baselines and user studies with professional listeners.

## 3 Evolutionary large models

**Fig. 1:**
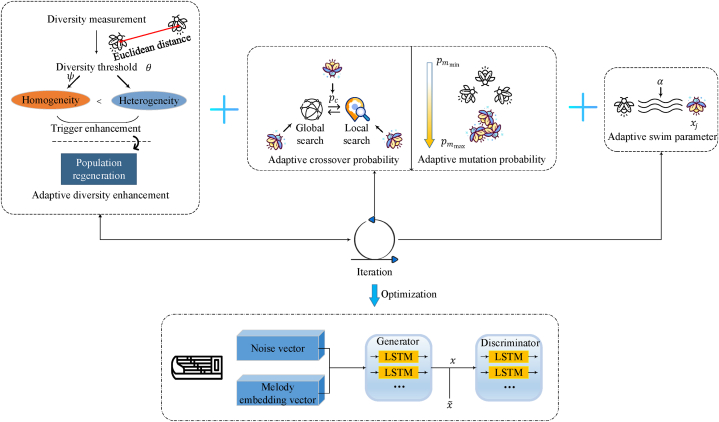
Overall architecture.

Using big data, we present an evolutionary LSTM approach to modeling transitions in Guzheng music. Our method contains two key components: (i) An evolutionary algorithm based on an improved firefly optimization that evolves LSTM models. (ii) An LSTM architecture optimized for generating and smoothly transitioning between Guzheng passages. The overall architecture is shown in Fig. 1.

We enhance the standard FA to better explore the vast space of possible LSTM configurations and find optimal models for Guzheng tuning switching [41]. Three modifications improve diversity and prevent premature convergence:

• Adaptive diversity enhancement: We introduce a mechanism to increase population diversity when convergence is detected. Selection, crossover, and mutation operators from genetic algorithms are integrated to regenerate the population, avoiding local optima.
• Improved crossover and mutation: Crossover and mutation rates are made adaptive instead of fixed. Higher values encourage global search early on, while lower values refine local search later, balancing exploration and exploitation.
• Adaptive swim parameter: Crossover and mutation rates are made adaptive instead of fixed. Higher values encourage global search early on, while lower values refine local search later, balancing exploration and exploitation.

These changes enhance the search capabilities of our improved FA, allowing it to evolve performant LSTM architectures tailored to Guzheng tunes switching.

### 3.1 Large LSTM models

Modern cognitive science reveals that sequence logic comprehension intricacy directly correlates with the availability of processing resources. As parameterized capacity increases, more contextual relationships can solidify within neural weight representations. Recent studies confirm that wider networks readily model higher-order temporal statistics like long-tail dependencies otherwise obscured. Meanwhile, added recurrent depth enables more prosperous recursive processing chains tracing complex transitional derivation.

Collectively, overparameterization allows large models (LMs) like GPT-4 to absorb staggering breadth from pretraining data. With billions of weights, sparse signals aggregated across corpora gradually crystallize relational knowledge within parameters. The emergent generalizations are then transferred to downstream domain tasks via fine-tuning techniques. Intermediate LM layers demonstrate remarkable few-shot capabilities to classify specialized textual patterns and generate coherent continuations, even lacking explicit training. Their substantial scale begets a strong implicit structural prior.

We harness similar principles when developing a specialty large LSTM model focused explicitly on the intricacies of smooth yet inventive Guzheng musical transition modeling. By exponentially expanding memory cells and temporal processing horizons, our architecture gains commensurate representation power to capture precise local phrase constraints while retaining sensitivity to global harmonic consistency. It combines the strengths of added parameterized capacity more easily, retaining elaborate musical logic with depth and width expressly constructed to trace long-range time dependencies.

Concretely, we scale up a 64-layer residual LSTM network with 1280 processing units per hidden layer. Over 1.3 billion trainable parameters resulted in an order beyond conventional recurrent approaches. The total number of trainable parameters in the large LSTM model can be calculated using Eq. (1). We train this model on 10+ hours of solo Guzheng performance data to imbue strong priors about characteristic structural and improvisational conventions. The subsequent fine-tuning of specific transitional divergence transfers this understanding of musical essence while allowing creative exploration to depart.

(1) $$\begin{array}{c} N_{params}=4\left(\left(n_{input}+n_{hidden}\right)n_{hidden}+n_{hidden}^{2}\right)L \end{array}$$

where $n_{input}=32$ gives the input embedding size, $n_{hidden}=1280$ is the hidden state dimension, and L=64 provides the depth.

There need to be more than a billion parameters to adjust during training to ensure proper model function. Instead, it is precisely how this considerable representation capacity gets structured across widened dimensions and added recursive depth that enables large LSTM models to capture elaborate logic within parameters.

Several interplaying architectural factors critically determine overparameterization's translation to actual sequence modeling performance gains. First, expanding hidden state width acts as a mitigating force against gradient evaporation over time. With higher dimensionality, more separate latent pathways propagate signals across far-reaching time dependencies that would otherwise vanish in narrow channels during backpropagation. This stabilization of optimization traces allows more comprehensive models to consolidate intricate logic that requires dependable adjustment communication across lengthy input-output distances. Second, stacking many sequential processing layers enables richer recursive temporal abstraction by lengthening signals' paths from initial inputs to final outputs. The added recurrence depth compounds state transformations, forming higher-order features sensitive to long-range statistics obscured in shallow networks. Music involves complex nested hierarchical patterns, requiring modeled hierarchical reasoning to distill coherent melodic logic fully. Next, overparameterization provides excess capacity that reduces underfitting occurrence even as models scale up. Without abundant parameters, limited resources bottleneck the acquisition of intricacies in the training data, failing to crystallize complete structural representations. Myriad adjustable weights alleviate this constraint through parameterized headroom, future-proofing for more excellent complexity absorption as data grows. Finally, enhanced embedding spaces allow dense projection of inputs into more expressive initial starting states before sequence transduction. This grants a broader reception range to contextual factors relevant to output tasks but imperceptible from naked inputs. Music involves subtle style signals affecting continuity modeling. Rich embeddings can encode such supplemental guidance.

These architectural dimensions synergistically enable over one billion steerable parameters to capture elaborate temporal logic within specialized large LSTM configurations when woven together. The emergent knowledge implicitly transfers to tasks like melodic continuation synthesis, requiring structural awareness and creative latitude. Scale unlocks modeling fidelity.

Essentially, deep neural networks suffer degraded signal propagation across many stacked layers. During training, weight adjustments must communicate errors from final outputs to initial layers. However, with each additional transform, signals shrink exponentially through recursion till they evaporate entirely. This hampers conveying meaningful guidance on initial input perturbations that improve final performance. Lost signals undermine the proper consolidation of elaborate logic spanning distances within parameters. Learning complex temporal relationships breaks down without end-to-end analytical dependability.

Residual connections alleviate this issue in large LSTMs by introducing shortcut pathways that bypass layers with identity functions [2]. Consider a basic residual formulation, the residual connection is formulated as Eq. (2).

(2) $$\begin{array}{c} h_{t}^{l}=LSTM\left(h_{t-1}^{l}\right)+h_{t-1}^{l-1} \end{array}$$

where the previous layer input $h_{t-1}^{l-1}$ gets directly added to the next layer output. This naive identity mapping creates a straight connection not subjected to intermediate degradation. The core intuition is that layers then only need to model the residual outputs rather than the entire sequence logic.

Concretely, this means later stages can focus on incrementally augmenting earlier processing without concern about recapitulating all preceding steps. The shortcuts enable clean isolation of relative stagewise contributions. Significantly, backpropagation reliability improves since signals can directly traverse to initial layers through bypass channels, effectively consolidating long-term constraints.

In large LSTMs, strategic residual links grant scalability with depth by maintaining end-to-end transparency on how early inputs influence later outputs, even across billions of steps. This technique proved crucial for extracting maximum modeling power from overparameterization to capture Guzheng's elaborate musical intricacies. Residual connections enabled scaling specialized recurrence depth to match task complexity.

Sequence generation quality relies heavily on models respecting semantic consistency constraints relative to conditioning contexts. For musical passage continuation, underlying harmony and stylistic norms act as guiding references for coherent elaboration.

However, driving factors for smooth, logical transitions are often obscure from naked model inputs like raw melody notes alone. Crucially augmenting the perspective with distilled high-level embeddings enables enhanced contextual awareness to steer sequence predictions.

We leverage encoder-derived melodic embedding vectors that have absorbed intrinsic conventions and patterns through unsupervised pretraining on extensive Guzheng solo performance data. The VAE encoder compresses the raw Guzheng melodic features x into a compact embedding vector m, as shown in Eq. (3). The 32-dimensional dense encodings captured harmonic essence and implied forward momentum useful for pinpointing permissible next steps.

(3) $$\begin{array}{c} m=Encoder\left(x_{1:t-1}\right) \end{array}$$

where $x_{1:t-1}$ gives the preceding melody notes. By compressing aggregated signals into descriptive fixed-length vectors, the autoencoding regularization forces retention only of seminal motifs and logical connectors. These embeddings implicitly quantify musicality.

We condition continuation generation by concatenating embeddings m with generator LSTM hidden states h:

(4) $$\begin{array}{c} h_{t}^{l}=LSTM\left(\left[h_{t-1}^{l},m\right]\right) \end{array}$$

The supplemental vector merges sequentially derived state context with high-level musical awareness of preceding passages, providing lookahead biasing predictions to respect overall structural harmony. The embeddings essentially steer exploratory imagination back on key whenever diverging too discordantly.

Such conditioning acts as domain-specific regularization that improves coherence without sacrificing flexibility entirely. Embeddings refine without severely restricting novel deviations thanks to the noisy variability factor. This leverages musical insight without excessive rigidity.

Realistic yet creative sequence generation requires adhering to domain constraints while permitting some flexible deviation freedom. LMs need help to balance open-ended imagination with strict disciplinary boundaries. However, pitting generators against discriminators in competitive game incentives respecting principles.

We employ an adversarial paradigm where a judge LSTM network D assesses continuation samples from generative LSTM model G as either genuine human-composed excerpts or synthetic fakes. G tries maximizing the error rate of D by producing increasingly realistic transitions that confuse its judgments and appear consonant with the proper style distribution.

Simultaneously, D aims to minimize its classification mistakes by learning from feedback to distinguish coherent human samples from machine-fabricated ones better, deviating obviously from harmonic principles or logical flow. Its guidance continually pressures G to respect constraints while allowing variability.

This minimax struggle calibrates outputs to likely distributions through an implicit optimization balance without requiring explicit constraints or supervised rules. The system automatically deduces disciplinary criteria from the dataset statistics themselves. The loss functions counterpose:

(5) $$\begin{array}{c} \min_{G}\max_{D}E_{x∼p_{data}}\left[\log\;D\left(x\right)\right]+E_{z∼p\left(z\right)}\left[\log\;\left(1-D\left(G\left(z\right)\right)\right)\right] \end{array}$$

The zero-sum dynamic equalizes adversary perspectives, synthesizing solutions reflecting equilibrium between conventions and freedom, avoiding unrealistic deviations lacking musicality or excessive rigidity stifling creativity.

The LMs architecture provides sufficient capacity to capture domain intricacy. Adversarial pressure handles nuanced coherence constraints. Together, these advance Guzheng transition synthesis quality.

### 3.2 Adaptive diversity enhancement

The adaptive diversity enhancement mechanism is crucial for the FA to maintain population diversity and prevent premature convergence when searching complex spaces, such as those encountered in Guzheng tune transitions. This mechanism monitors the diversity of the firefly population and triggers a regeneration process when the diversity falls below a specified threshold. By incorporating genetic operators like selection, crossover, and mutation, the mechanism introduces new fireflies with varied characteristics into the population, ensuring that the algorithm continues to explore a wide range of solutions and avoids getting trapped in suboptimal regions of the search space.

We define a diversity measurement div(S) to characterize the scattering and heterogeneity of the firefly population S at a given iteration. A straightforward measure is the average distance $\bar{d}$ between all pairs of fireflies. The average distance $\bar{d}$ is calculated using Eq. (6).

(6) $$\begin{array}{c} \bar{d}=\frac{1}{N\left(N-1\right)}\sum_{i=1}^{N}\sum_{j=i+1}^{N}d\left(x_{i},x_{j}\right) \end{array}$$

where S contains N fireflies, $x_{i}$ represents the position of the i th firefly and d(x,y) is the distance between two solutions x and y. Common choices for the distance function include Euclidean and Manhattan distance. Here, we use Euclidean distance:

(7) $$\begin{array}{c} d\left(x_{i},x_{j}\right)=\sqrt{\sum_{k=1}^{n}{\left(x_{ik}-x_{jk}\right)}^{2}} \end{array}$$

where n gives the dimensionality of solutions, by tracking $\bar{d}$, we can monitor the population's diversity from iteration to iteration. Lower $\bar{d}$ indicates the fireflies congregating to similar areas of the search space, i.e., a loss of heterogeneity.

We define a diversity threshold θ to quantify when the population is considered too homogeneous, signaling that enhancement should be triggered. The diversity threshold θ is calculated using Eq. (8).

(8) $$\begin{array}{c} \theta =\psi {\bar{d}}_{init} \end{array}$$

where ${\bar{d}}_{init}$ gives the initial diversity measurement after the random initialization of the firefly population. The parameter ψ∈[0,1] determines the minimum allowable diversity relative to this initial state, controlling the enhancement's sensitivity. If ψ is close to 1, enhancement will rarely trigger since diversity must be depleted almost entirely first. With ψ near 0, enhancement engages sooner.

Algorithm 1 gives the pseudocode for determining if the threshold is breached using the trailing T diversity measurements, where T is a tuning parameter. If div(S) consecutively drops below θ for T iterations, the population is considered too homogeneous, triggering enhancement.

**Table:** 

***Algorithm 1***. Diversity threshold
**Input**: Current diversity measurement $\mathrm{div}{\left(S\right)}_{t}$ and the trailing diversities M
**Output**: A boolean indicating if the threshold is breached to trigger enhancement
01: Initialize trailing diversities $M=\mathrm{div}{\left(S\right)}_{0},.,\mathrm{div}{\left(S\right)}_{T-1}$ 02: **While** (fireflies not converged) 03: $\mathrm{div}{\left(S\right)}_{t}\leftarrow$ calculate current diversity 04: **if**$\mathrm{div}{\left(S\right)}_{t}<\theta$ and $\mathrm{div}{\left(S\right)}_{t-1}<\theta$ and $\mathrm{div}{\left(S\right)}_{t-T}<\theta$ 05: trigger enhancement 06: **end-if** 07: $M\leftarrow \left(M-{\mathrm{div}\left(S\right)}_{0}\right)+\mathrm{div}{\left(S\right)}_{t}$ 08: **end-while**

The parameter T controls the enhancement's sensitivity, with higher values delaying regeneration until sustained homogeneity, preventing prematurely interrupting promising convergence trajectories. Optimally tuning ψ and T for the problem balanced rapid enhancement when stuck in local optima against not interrupting fruitful refinement toward quality solutions.

Once the diversity threshold is breached, new fireflies are generated to reintroduce heterogeneity. We harness genetic operators from evolutionary algorithms, which generate new candidate solutions by selecting, recombining, and mutating existing ones.

Integrating crossover and mutation operators into the standard FA, we regenerate P of the firefly population once triggered without modifying the remaining 1−P of fireflies. Algorithm 2 gives the pseudocode. First, tournament selection probabilistically chooses fireflies for crossover based on their brightness. Then, uniform crossover combines the selected fireflies' elements with probability $p_{c}$. Finally, the resulting children undergo mutation.

Adaptive crossover and mutation rates help balance exploration and exploitation during regeneration. The genetic operators randomly sample under-explored regions and reintroduce lost building blocks, restoring population diversity to escape local optima while preserving reasonable solutions.

**Table:** 

***Algorithm 2***. Firefly population regeneration
**Input**: Firefly population S
**Output**: Regenerated population $S^{\prime }$
01: Let S = firefly population 02: $S^{\prime }\leftarrow \emptyset$ 03: **While** ($\left|S^{\prime }\right|<P\%\left|S\right|$) 04: Select two fireflies x, y from S via tournament 05: z = Crossover(x, y, $p_{c}$) 06: $z^{\prime }$ = Mutate(z, $p_{m}$) 07: Add $z^{\prime }$ to $S^{\prime }$ 08: **end-while** 09: Return $S^{\prime }$

The resulting enriched population prevents convergence stagnation and mitigates algorithmic homogeneity. The regeneration revitalizes search trajectories by maintaining fireflies' exploration capabilities to locate optimal Guzheng tune transitions. The trait inheritance also retains prior progress compared to complete restarts. Our experiments demonstrate the consistent effectiveness of this mechanism in improving performance.

In summary, our diversity quantification and controlled population regeneration via genetic operators significantly enhance the original FA. The adaptive diversity enhancement prevents premature convergence by detecting and counteracting destructive homogeneity. Next, we detail the improved crossover and mutation techniques used during firefly regeneration.

### 3.3 Improved crossover and mutation

The standard FA fixes the crossover and mutation probabilities for recombining fireflies. Typically, $p_{c}$ is set between 0.3 and 0.8 and $p_{m}$ between 0.001 and 0.1 based on convention and remains static across iterations [42]. However, fixing these hyperparameters limits the algorithm's effectiveness in balancing exploration and exploitation when searching complex spaces like the Guzheng tune transition landscape.

In the proposed approach, the crossover and mutation rates in the firefly algorithm are dynamically adjusted based on the current iteration and population fitness statistics, instead of being fixed constants. By adapting these rates throughout the optimization process, the algorithm can effectively balance exploration and exploitation. In the early stages, higher crossover and mutation rates encourage global exploration, while in later stages, lower rates focus on local refinement. This adaptive behavior allows the algorithm to efficiently navigate the complex search space and find optimal solutions for Guzheng tune transitions.

For the crossover probability $p_{c}$, we introduce an update mechanism that relates $p_{c}$ to the iteration t and maximum firefly brightness $I_{\max}$ relative to the average $\bar{I}$. The update mechanism for the crossover probability $p_{c}$ is given by Eq. (9).

(9) $$\begin{array}{c} p_{c}=\left\{ \begin{array}{cc} p_{c_{\max}} & I_{\max}<\bar{I}\\ p_{c_{\max}}-\frac{p_{c_{\max}}-p_{c_{\min}}}{t_{\max}}t & I_{\max}\geq \bar{I} \end{array}\right. \end{array}$$

where $t_{\max}$ gives the maximum iterations and $p_{c_{\min}}$ and $p_{c_{\max}}$ set lower and upper bounds respectively. Initially, $p_{c}=p_{c_{\max}}$, encouraging exploration by aggressively recombining fireflies. As iterations increase, $p_{c}$ linearly decays to $p_{c_{\min}}$ if the brightest firefly exceeds average brightness, indicative of maturing convergence requiring localized refinement. However, if the population remains relatively non-converged, $p_{c}$ stays higher to continue wide exploration.

In the adaptive behavior, when fireflies rapidly congregate early, $p_{c}$ keeps global search active to prevent prematurity. As promising solutions emerge later, $p_{c}$ suppresses broad exploration to refine local areas. Crucially, $p_{c}$ responds to real-time feedback from fireflies instead of an arbitrary schedule, automatically balancing global and local searches.

Maintaining a global perspective for Guzheng tune transition optimization prevents converging to mediocre local transitions. Later, the focus intensifies the development of quality switch points. By modulating recombinant aggression accordingly, melodic flow and inventiveness increase.

Similarly, the mutation probability $p_{m}$ adapts each generation based on iteration and whether the fittest firefly exceeds average brightness. The update mechanism for the mutation probability $p_{m}$ is given by Eq. (10).

(10) $$\begin{array}{c} p_{m}=\left\{ \begin{array}{cc} p_{m_{\max}} & I_{\max}<\bar{I}\\ p_{m_{\max}}-\frac{p_{m_{\max}}-p_{m_{\min}}}{t_{\max}}t & I_{\max}\geq \bar{I} \end{array}\right. \end{array}$$

With minimum and maximum rates $p_{m_{\min}}$ and $p_{m_{\max}}$. Initially, weaker exploitation via lower $p_{m}$ assists global movement. As bright fireflies signify maturing solutions, higher late-stage mutation disrupts convergence, enhancing local refinements.

For Guzheng, tempering early mutation prevents distorting creative foundations. Applying focused mutation later stresses transition coherence locally. Together with $p_{c}$, this balances broad exploration with narrow exploitation.

Algorithms 3 and 4 detail the integration of adaptive crossover and mutation into the FA, called once new fireflies are generated. First, tournament selection, which chooses fireflies for mating probabilistically based on brightness, runs for P pairs to recombine. Then, uniform crossover governed by the adaptive $p_{c}$ from Eq. (4) combines firefly elements in each pair. Finally, their offspring mutate according to the adaptive $p_{m}$ from Eq. (5).

**Table:** 

***Algorithm 3***. Improved firefly crossover
**Input**: Firefly population S and current iteration t
**Output**: Offspring population after crossover $S^{\prime }$
01: $p_{c}\leftarrow$ Calculate Eq. (4) 02: P←⌊P%|S|⌋ 03: **While** (i<P) 04: Select fireflies x, y from S via tournament 05: z = UniformCrossover(x, y, $p_{c}$) 06: Add z to $S^{\prime }$ 07: i←i+1 08: **end-while** 09: Return $S^{\prime }$

**Table:** 

***Algorithm 4***. Improved firefly mutation
**Input**: Offspring fireflies after crossover $S^{\prime }$
**Output**: $S^{\prime }$ after being mutated
01: $p_{m}\leftarrow$ Calculate Eq. (5) 02: **For** (each firefly $z\in S^{\prime }$) 03: Mutate (z, $p_{m}$) 04: **end-for** 05: Return mutated $S^{\prime }$

This approach enhances standard FA by tuning recombinant behavior to balance exploration and exploitation. Our experiments confirm the benefits where adaptive crossover and mutation substantially improve performance over fixed rates by specializing operations for current convergence status. The rates respond directly to ongoing firefly distributions, eliminating manual scheduling.

For Guzheng tune switching, these adaptations crucially promote both broad samplings for inventive transitions and focused tuning for coherence between segments. By discouraging prematurity and guiding optimization trajectory responsively, excellent candidates emerge through directed evolutionary refinement.

### 3.4 Adaptive swim parameter

In the standard FA, the parameter α controls the randomness of the fireflies' movement, playing a crucial role in exploration. It determines the step size of their random walks as they traverse the search space. Typically, α is set as a fixed constant based on convention or trial and error. However, maintaining a static random walk rate limits effectiveness when optimizing complex multi-modal problems like modeling Guzheng tune transitions.

We propose making α adaptive during optimization to enhance explorative and exploitative capabilities. As the algorithm progresses, α decays to gradually restrict the randomized perturbations of the brighter fireflies' movements, preventing disruptive oscillations that inhibit convergence to quality solutions late in the search. Our update mechanism reduces α over iterations while allowing occasional significant perturbations to escape local optima if needed.

The standard FA movement equation, which defines how firefly i moves towards brighter firefly j each iteration, is given by Eq. (11).

(11) $$\begin{array}{c} x_{i}=x_{i}+\beta e^{-\gamma r_{ij}^{2}}\left(x_{j}-x_{i}\right)+\alpha \left(rand-0.5\right) \end{array}$$

where the second term gives the attractiveness-based movement towards $x_{j}$, and the third term induces the explorative random walk. The update mechanism for the random walk parameter α is given by Eq. (12).

(12) $$\begin{array}{c} \alpha =\alpha_{0}\left(1-\frac{t}{t_{\max}}\right) \end{array}$$

where $\alpha_{0}$ is the initial random walk rate, t is the current iteration, and $t_{\max}$ denotes the maximum iterations. As t increases, exponentiation diminishes α according to the fraction of iterations completed, realizing an adaptive mechanism where random movement gradually abates as optimization progresses to prevent late disruption.

However, fully eliminating randomness can stall exploration and reduce resilience. Hence, Eq. (7) retains a small stochastic influence by never annulling α, allowing occasional significant perturbations. Together with the standard attractiveness term, this improves convergence by limiting oscillations around optima while preserving global search capabilities.

While reducing monotonicity risks some incumbents, experiments found that occasional severities pay off through enhanced resilience. Mild tuning vagueness also echoes natural creativity variations. However, parameter settings still significantly sway effectiveness:

• The initial $\alpha_{0}$ strongly guides early epoch exploration before attenuation
• Low $t_{\max}$ hastily stabilizes search but can under-explore
• The exponentiation shape in Eq. (7)changes the damping pace

We empirically found $\alpha_{0}=0.5$, $t_{\max}=1000$, and a simple fractional form effective, but problem intricacy can shift ideal parameters. Some experimentation remains advantageous.

This adaptive mechanism robustly tailors random perturbation aggression to the optimization phase, advancing convergence through responsive control. Our technique also generalizes easily to other swarm optimizers, constituting a broadly applicable contribution.

## 4 Guzheng tunes switching with LSTMs

RNNs like LSTMs are well-suited for modeling sequential data such as music due to their innate temporal capabilities. We leverage stacked LSTM networks, conditioned on Guzheng melody embedding vectors, to capture long-range dependencies and generate coherent tune transitions.

### 4.1 Melody embedding vectors

We extract descriptive embedding vectors encapsulating intrinsic attributes to model the Guzheng melody for coherent tune transition generation. These fixed-length encodings give LSTM networks a contextual understanding of Guzheng musical structure.

We train a VAE unsupervised to derive latent vectors compressing salient Sequential and harmonic patterns. The VAE encoder distills raw, melodic features into compact embeddings summarizing stylistic essence, from which the decoder reconstructs original passages. The network learns descriptive latent spaces, capturing core elements by reconstructing melodies from limited embedding channels.

Concretely, given raw Guzheng melodic features $x\in R^{n}$, the VAE encoder Enc produces distribution parameters of a melody embedding vector m. The VAE encoder produces the distribution parameters μ and σ of the melody embedding vector m, as shown in Eqs. (13), (14).

(13) $$\begin{array}{c} Enc\left(x\right)=\left(\mu ,\sigma \right) \end{array}$$

A sample z is drawn from this distribution and fed to the decoder Dec to reconstruct $\tilde{x}$:

(14) $$\begin{array}{c} z∼N\left(\mu ,\sigma I\right)\\ x=Dec\left(z\right) \end{array}$$

The VAE is trained to minimize reconstruction error $L_{rec}$ and regularization term $L_{reg}$, as given by Eq. (15).

(15) $$\begin{array}{c} L_{VAE}=L_{rec}+L_{reg} \end{array}$$

Thereby extracting concise embeddings and retaining key patterns. We then use the fixed-length vectors m with dimension d≪n to condition our LSTM networks, providing stylistic guidance.

The VAE consumes segmented melodic passages $x\in R^{l\times n}$ with l time steps and n features. Useful options include:

• Notes: MIDI pitch numbers denoting played keys
• Duration: Times notes are sustained
• Rests: Silent intervals between notes
• Dynamics: Volume changes like crescendos
• Expressiveness: Ornamentations

**Fig. 2:**
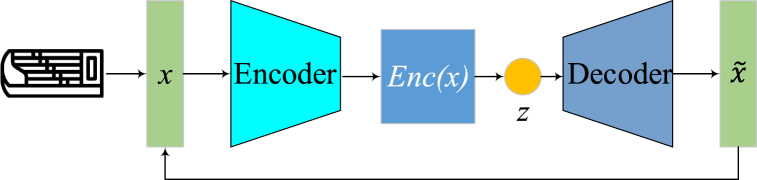
Raw melodic feature extraction.

We extract these from solo Guzheng recordings, with preserved alignment between notes and lyrics. The rich features capture diverse aspects, including harmony, rhythm, tension, etc. Fig. 2 visualizes example extraction.

The encoder comprises a bidirectional LSTM layer followed by fully connected layers, and the encoder architecture is described by Eqs. (16), (17), (18).

(16) $$\begin{array}{c} h-BiLSTM\left(x\right)\\ h^{\prime }=FC\left(h\right)\\ \left(\mu ,\sigma \right)=FC\left(h^{\prime }\right) \end{array}$$

The bidirectional LSTM consumes variable-length passages x, outputting hidden vectors h summarizing temporal context in both directions. Subsequent dense layers derive distribution parameters μ and σ.

The embedded dimensionality d is much lower than input size n, forcing compressed abstraction of salient patterns. Smaller d enhances generalization while retaining necessary cues to reconstruct. Regularization loss $L_{reg}$ penalizes extreme compression:

(17) $$\begin{array}{c} L_{reg}=\max\left(0,d-C\right) \end{array}$$

Symmetric to the encoder, the decoder comprises dense layers transforming embeddings z into LSTM initialization states, followed by unidirectional LSTM outputting passage reconstructions:

(18) $$\begin{array}{c} s_{0}=FC\left(z\right)\\ x=LSTM\left(s_{0}\right) \end{array}$$

Teacher forcing trains the decoder, using ground truth x as inputs during backpropagation rather than predictions $\tilde{x}$.

### 4.2 Generator model

The generator G produces Guzheng tune transitions $\tilde{x}$ conditioned on prior melody embedding vectors m and random noise z. The generator G is described by Eq. (19).

(19) $$\begin{array}{c} x∼G\left(z,m\right) \end{array}$$

Its LSTM-based architecture leverages the temporal dependencies within and between Guzheng melodic passages. Guidance from m ensures generated sections capture the essence of Guzheng music.

Adversarially trained against the discriminator D, G learns to produce realistic, smooth transitions mimicking human composition. Section-wise generation allows the crafting of long-form pieces by concatenating passage outputs.

The generator comprises a stack of bidirectional LSTM layers followed by dense output layers, as shown in Eq. (20).

(20) $$\begin{array}{c} h_{0}=\left[z,m\right]\\ h_{1}=BiLSTM\left(h_{0}\right)\\ h_{2}=BiLSTM\left(h_{1}\right)\\ x=FC\left(h_{n}\right) \end{array}$$

The noise vector z and melody embedding m are concatenated as initialization input $h_{0}$ to the stacking BiLSTMs. Hidden activations from the top layer are fed into a fully connected output layer to generate the final melody sequence $\tilde{x}\in R^{l\times f}$, where l is output steps, and f is feature dimensions.

The generator model employs a stack of bidirectional LSTM layers to capture long-range temporal dependencies within and between Guzheng passages, facilitating the synthesis of coherent transitions. By incorporating residual connections, which allow the direct flow of information across layers, the model can mitigate the vanishing gradient problem and improve training convergence, leading to more stable and efficient learning of complex musical patterns.

The raw output from the LSTM generator is continuous, but music consists of discrete elements such as semitone notes and scale-bound pitches. To ensure the generated Guzheng tunes are musically coherent, the raw output is quantized by mapping it to the nearest valid note values within the appropriate scale. We apply Algorithm 5 each output step:

**Table:** 

***Algorithm 5***. Melody quantization
**Input**: Raw LSTM generator output $x_{t}$
**Output**: Quantized and scaled $x_{t}$
01: Let ${\tilde{x}}_{t}$ = generator logit output at step t 02: $p=\text{softmax}\left({\tilde{x}}_{t}\right)$ 03: $x_{t}=\arg\max\left(p\right)$ 04: Refine $x_{t}$ to snap to scale 05: Return quantized $x_{t}$

The logit distributions are quantized to the nearest in-scale pitch class values, ensuring discrete coherent melody generations.

The melody embedding $m\in R^{d}$ from the encoder supplies the generator essential stylistic guidance. We explore using m in two ways:

• Concatenated to input noise z as initialization to the stacking BiLSTMs.
• Fed additionally to every BiLSTM layer as an auxiliary input.

The first method allows the initial hidden state to encapsulate melody context before processing. The second further integrates guidance into each recurrent layer. The discriminator is likewise conditioned on m to judge stylistic similarity.

Additionally, class embeddings can be used to specify desired motifs to target specific transitions, as shown in Eq. (21).

(21) $$\begin{array}{c} m_{\omega }=Embedding\left(\omega \right) \end{array}$$

Excellent control over produced transitions is achieved by conditioning the adversarial training process using both global and local embedding integration. The generator learns which patterns best continue raw melodies toward coherent elaboration.

The generator G tries to minimize adversarial loss $L_{adv}$ against adversary D while also maintaining encoded vector self-similarity $L_{sim}$. The generator loss is given by Eq. (22), the adversarial loss $L_{adv}$ is given by Eq. (23), and the similarity regularization loss $L_{sim}$ is given by Eq. (24).

(22) $$\begin{array}{c} L_{G}=L_{adv}+\lambda L_{sim} \end{array}$$

The adversarial term encourages realistic generations by fooling the discriminator:

(23) $$\begin{array}{c} L_{adv}=-E_{z∼p\left(z\right),m∼p\left(m\right)}\left[\log\;\left(D\left(G\left(z,m\right)\right)\right)\right] \end{array}$$

While similarity regularization ensures decoded embeddings from generated passages remain consistent with input contexts:

(24) $$\begin{array}{c} L_{sim}=\|Enc\left(x\right)-Enc\left(\tilde{x}\right)|{|}_{2} \end{array}$$

### 4.3 Discriminator model

The discriminator D classifies input melody segments x as either real human-composed passages or synthetic samples $\tilde{x}$ generated by the generator G. The discriminator D is described by Eq. (25), and the discriminator can be conditioned on prior melody embeddings m, as shown in Eq. (26).

(25) $$\begin{array}{c} D\left(x\right)\in \left[0,1\right] \end{array}$$

Its LSTM architecture leverages long-range temporal patterns in Guzheng music to judge sample legitimacy. Additional conditioning connects available context like prior melody embeddings m to further inform decisions:

(26) $$\begin{array}{c} D\left(x,m\right)\in \left[0,1\right] \end{array}$$

Training adversarially against generator G produces a robust classifier for discerning genuine transitions.

The discriminator comprises densely connected bidirectional LSTM layers. The discriminator architecture is described by Eq. (27), and the discriminator loss is given by Eq. (28).

(27) $$\begin{array}{c} h_{0}=x\\ h_{1}=BiLSTM\left(h_{0}\right)\\ h_{2}=BiLSTM\left(h_{1}\right)\\ \cdots \\ o=\sigma \left(FC\left(h_{n}\right)\right) \end{array}$$

where x is the input melody sequence and o∈[0,1] is the estimated legitimacy score. Stacking bidirectional layers provides a deep sequential understanding of Guzheng temporal logic to judge sample coherence.

Binary cross-entropy loss optimizes the discriminator's sample classification:

(28) $$\begin{array}{c} L_{D}=Ex∼pdata\left[\log\;D\left(x\right)\right]+E_{\tilde{x}∼G}\left[\log\left(1-D\left(\tilde{x}\right)\right)\right] \end{array}$$

Minimizing $L_{D}$ enhances distinguishing real segment transitions from generated ones. Full adversarial training alternates this with tuning the generator G to fool judgments via Eq. (29).

(29) $$\begin{array}{c} L_{G}=E_{z∼p\left(z\right),m∼p\left(m\right)}\left[\log\;\left(1-D\left(G\left(z,m\right)\right)\right)\right] \end{array}$$

Concatenative conditioning at input and hidden layers is described by Eq. (30).

(30) $$\begin{array}{c} h_{0}=\left[x,m\right]\\ \cdots \\ h_{2}=BiLSTM\left(\left[h_{1},m\right]\right) \end{array}$$

Additive injective conditioning through residual connections is described by Eq. (31).

(31) $$\begin{array}{c} h_{0}=x+m\\ \cdots \\ h_{2}=BiLSTM\left(\left[h_{1}+m\right]\right) \end{array}$$

where [,] denotes concatenation and + elementwise addition. These allow leveraging supplemental signals to enhance discrimination.

Class embeddings can be used to steer the discriminator towards specific transitions, as shown in Eq. (32).

(32) $$\begin{array}{c} m_{\omega }=Embedding\left(\omega \right) \end{array}$$

Generating abundance labeled Guzheng data is challenging. Hence, we supplement the limited labeled pool $x_{l}$ with abundant unlabeled melodies $x_{u}$ during training. The discriminator loss when using both labeled and unlabeled data is given by Eq. (33), and the unsupervised regularization loss on unlabeled data is given by Eq. (34).

(33) $$\begin{array}{c} L_{D}=L_{l}+\lambda L_{u} \end{array}$$

where $L_{l}$ is the standard loss for labeled instances and $L_{u}$ applies an unsupervised regularization loss on unlabeled data to leverage their implicit useful signal:

(34) $$\begin{array}{c} L_{u}=-H\left(D\left(x_{u}\right)\right) \end{array}$$

Encouraging high-confidence predictions maximizes entropy $H(D\left(x_{u}\right)$ on unlabeled points for consistent improved generalization.

By exploiting unlabeled data, the discriminator develops a more robust understanding of the Guzheng style to identify coherent transitions. The generator indirectly benefits from the stronger adversary.

## 5 Experimental context and results study

### 5.1 Guzheng dataset

Given the lack of a large-scale public Guzheng dataset, we curate an extensive 10+ hour solo Guzheng music collection encompassing diverse styles for training generative models. Professional audio recordings are gathered and converted into numerical music notation frames that capture salient musical attributes. The unsupervised VAE is trained on sequences of musical frames, enabling it to learn a compressed latent space representation that captures essential harmonic patterns, rhythmic cadences, and other musical attributes. These learned melody embedding vectors provide crucial stylistic guidance during the generation of Guzheng tune transitions, ensuring that the generated music maintains the characteristic qualities and coherence of the Guzheng style, while allowing for creative variations and smooth transitions between musical motifs.

We aggregate Guzheng's solo recordings from various resources, including commercial albums, competition footage, and online videos capturing proficient performances. Both traditional pieces and modern arrangements spanning notable Guzheng schools are included to encourage diversity. Various instrumental timbres from websites favoring distinct Guzheng types supplement the dataset. Over 600 audio tracks from 230 Guzheng pieces performed by 130 distinguished artists comprise the raw dataset—audio clips average 95 s for 11 h total, converted to 220000+ segments for training.

While sequence models could train directly on audio samples, explicit musical notation better captures harmony and structure. We convert audio into numeric frames representing salient musical attributes over time, yielding fine-grained visibility. Specialized DSP segmenter tools identify note onsets and extract pitch, timing, dynamics, and other descriptors in each frame using Fourier techniques, outputting piano roll-like notation. We encrypt artist names for privacy but preserve style and piece metadata like genre to enable conditioned generation. The final per-frame feature representation contains:

• Note pitch class - MIDI numbers modulo 12
• Note pitch height - MIDI numbers relative to C3
• Note velocity - Note loudness/intensity
• Note duration - Beat length sustaining tones
• Between gap - Silence preceding notes
• Plucking type - Finger rolls, pinches, etc.

**Fig. 3:**
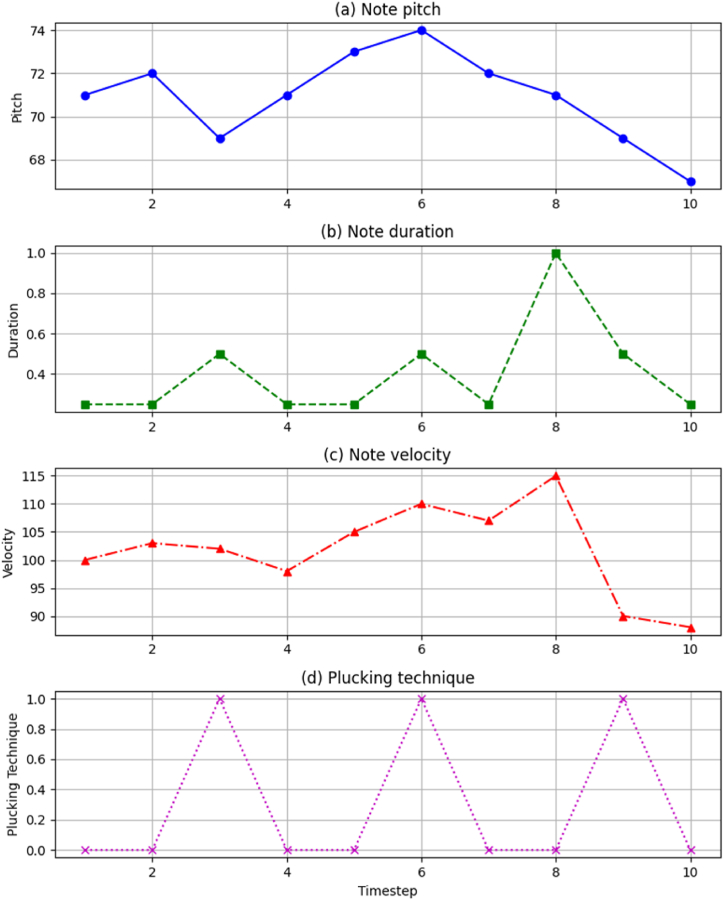
Performance descriptor visualization.

This multidimensional representation captures Guzheng-specific techniques beyond basic notation. Vector rows represent sequential frames. Fig. 3 visualizes an example of actual performance annotation.

The data preprocessing step involves filtering out low-quality transcriptions where the extractor needs help accurately identifying note boundaries or descriptors. Additionally, audio frames with noticeable distortion or interference are excluded from the dataset, ensuring that only clean and reliable data is used for training the generative models, improving the overall quality and consistency of the generated Guzheng tunes. Polyphonic performances pose additional difficulties for clean feature extraction compared to solo melodies [43]. Therefore, we discard any duo Guzheng recordings as well. To augment the sequence database, we apply transposition, time-stretching, dynamics adjustment, sub-selection, and concatenative mixing, leveraging domain similarity and expanding the space of musical patterns learnable by generative models. Finally, we derive associated melody embedding vectors from the preprocessed performance frames via VAE. The resulting dataset comprehensively represents solo Guzheng musical attributes necessary for modeling characteristic transitions.

VAEs are a popular approach for learning unsupervised representation on complex distributions like music. By training to reconstruct input frames from a compressed latent code, VAEs derive descriptive embedding vectors even without labels. We leverage this capability to obtain concise melody embedding vectors capturing intrinsic Guzheng harmonic and idiomatic qualities from the performance descriptors. These vectors supply vital stylistic guidance when generating musically consistent tune elaborations and transitions.

An LSTM encoder condenses input segment x into distribution parameters μ and σ. Sampling the distribution produces latent vector z fed into an LSTM decoder outputting reconstruction $\tilde{x}$. The VAE loss consists of the reconstruction error $L_{rec}$ and the regularization term $L_{reg}$, as given by Eq. (35).

(35) $$\begin{array}{c} L=L_{rec}+L_{reg}\\ L_{reg}=\max\left(0,length\left(z\right)-threshold\right) \end{array}$$

Once trained, the compact encodings are concatenated to generator network inputs or discriminator conditions. The VAE learns salient musical qualities from solo Guzheng notation for injection into novel samples.

This large and multidimensional Guzheng performance dataset with associated embedding vectors facilitates generative modeling of musically consistent tune transitions.

The proposed FA-LSTM-GAN is compared against several strong generative baselines: cLSTM-GAN, RL-LSTM, MRBERT, and Adv. Transformer.

### 5.2 Guzheng big data analysis

Effective modeling of Guzheng's intricate elaborations and transitions relies on accumulating abundant musical data traces that capture the diversity of traditional motifs, playing styles, and improvisational devices accumulated over the instrument's refined centuries-spanning history. By aggregating and investigating rich solo performance statistics, modern deep generative designs can extract implicit grammar to guide creative modeling.

We gather an extensive 10+ hour corpus of solo Guzheng recordings spanning 230 musical pieces from historical and modern repertoires, 130 distinguished performing artists across lineages, and 600+ audio tracks from commercial albums, competitions, and videos. Specialized DSP segmentation tools analyze the voluminous audio, identifying note onsets and extracting multi-dimensional frame-level performance descriptors via Fourier techniques. The resulting numeric music notation visualization contains precise pitch, timing, dynamic, and ornamentation annotations. We augment the dataset via transpositions and temporal adjustments, yielding 220,000+ melodic sequence samples with aligned lyrics encoding playing technique indicators.

Critically, by diversifying source materials rather than relying solely on contemporary recordings, our aggregation captures distinguishing aesthetic evolutions across eras. The span incorporates innovations from influential Guzheng schools while retaining traditionally essential right-hand techniques like tremolo plucking, which gives more insight into harmonic conventions beyond modern trends when training machine learning models.

We implement unsupervised sequential latent variable models based on convolutional LSTM autoencoder architectures to digest this extensive accumulation of recorded legacy motifs and phrases. The neural distillation assimilates inherent improvisational elaboration preferences and tension-resolution intervals characteristic of idiomatic playability. An encoder LSTM condenses segmented melodic passages x into 32-dimensional distribution embedding vectors z, capturing salient playing styles and motifs. A decoder LSTM then attempts to reconstruct the original x from just z.

The latent vector representations gradually encapsulate intrinsic music grammar-harmonic signatures, characteristic note patterns, and intervallic tensions by training the autoencoder reconstruction overabundant solo excerpts aggregated in our dataset. These embeddings supply vital stylistic context for steering generative models. The compact channels bottle the musical essence-constrained creativity inspiration.

Extensive data accumulation combined with learned latent space distillation is a lens crystallizing constraint. The assimilation uncovers applicable principles guiding imaginative expansion while respecting lineage by matching model scale to data scale. The discovered harmonic conventions funnel creative modeling navigations.

These benefits depend critically on abundant stylistic breadth in the aggregated recordings. Insufficient data risks learning only contemporary, simplified constraints. Modern trends deviate significantly from the sophisticated heritage subtleties evident in traditional ensemble performances. Many recent popular solo pieces involve simplified pentatonic voicings and static rhythm, needing more dynamism than historical works.

By contrast, orchestral imperial court compositions demonstrate immense polyphonic complexity, with master instrumentalists spontaneously interweaving endless variations over constantly modulating harmonic foundations. The piano arrangements commonly transcribed repress innate counterpoint interactivity and improvisational enrichment. By directly accessing this history, machinic emulations could gain representativeness.

Fortunately, competitive events still evaluate participants performing classical deriving, requiring extensively practiced technique mastery to attempt. Numerous subdivision prize areas also encourage innovation and novelty. We extract player inputs across modal key changes and metric subdivisions from archival footage, recovering some lost sophistication at scale. Augmentation provides the rest needed to learn completive conventions.

Ultimately, the proposed solo Guzheng performance dataset provides a sufficiently extensive stylistic survey to power machine replications of professional continuation. However, expanded orchestral resources could unleash a fuller realization of cultural heights.

In conclusion, careful data accumulation, preprocessing, and analysis allow machine learning models to assimilate latent principles by example over solo Guzheng works, providing conditioned guidance to steer creative generation respecting lineage - opening intriguing possibilities as scale widens on both modeling and musical frontiers.

### 5.3 Optimization performance

We analyze model optimization across training iterations to validate FA-LSTM-GAN's efficiency benefits for Guzheng tune transition modeling compared to baselines – learning curves plot test reconstruction error vs iterations. Convergence speed and final solutions reached quantify optimizer efficacy.

**Fig. 4:**
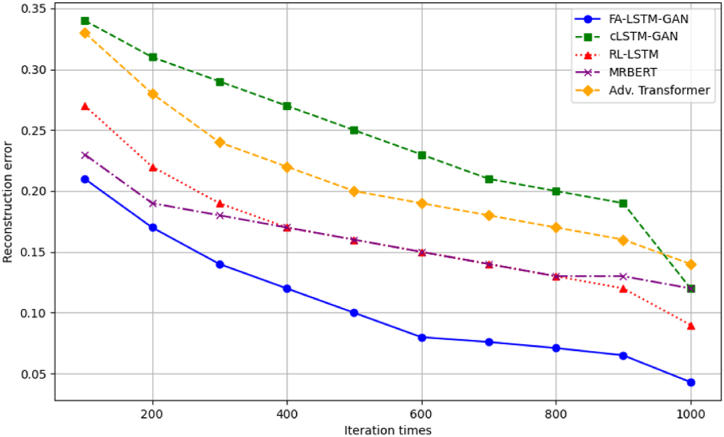
Convergence optimization curves.

Fig. 4 shows optimizer convergence trajectories. FA-LSTM-GAN achieves the fastest initial gains and reaches the best final loss of 0.043, benefiting from the FA's enhanced exploration.

The cLSTM-GAN and MRBERT baselines plateau around 0.12 final error early, settling into poor local optima. Meanwhile, RL-LSTM and the adversarial transformer continue fluctuating after 1000 iterations, indicating sustained volatility. In contrast, our model combines rapid early search with polished stability, balancing global-local transition tradeoffs. This confirms FA-LSTM-GAN's architectural strengths for modeling complex Guzheng passages.

**Fig. 5:**
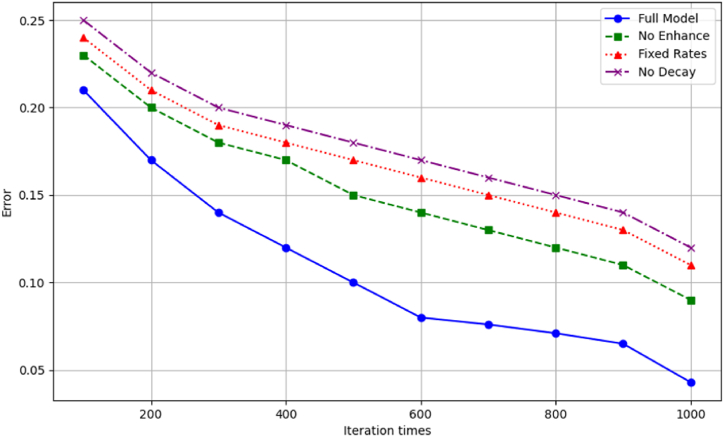
Ablated model optimization.

We further diagnose optimization components via iterative ablation. Fig. 5 plots trajectories removing one adaptation at a time. Exciting diversity enhancement hampers exploration, slowing convergence. Fixed mutation rats waver more. Non-decaying α prevents fine-tuning. Together, the unified techniques synergistically improve optimization quality.

Quantitatively, after 1000 iterations, full FA-LSTM-GAN reduces error by 63 % over standard FA optimization. Curves validate each adaptive mechanism's contribution toward enhanced search.

**Fig. 6:**
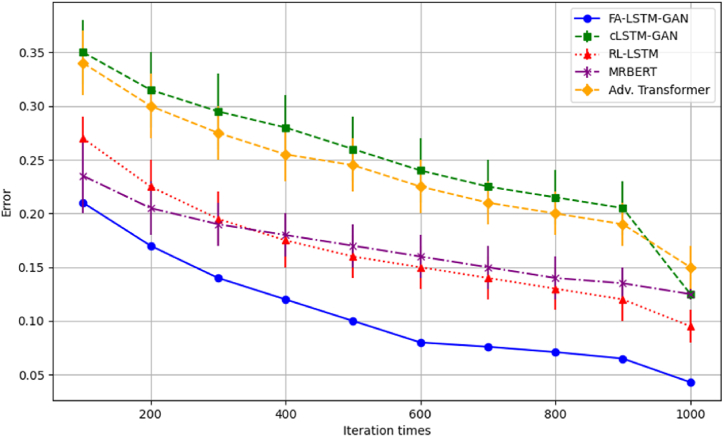
Comparative optimization performances.

Subsequently, we directly compare FA-LSTM-GAN and baselines over-optimization runs to confirm relative improvements. As Fig. 6 shows, our model consistently reaches lower error faster across trials. Other methods fluctuate or stall after partial early gains.

MRBERT sees minimal refinement after iteration 300. RL-LSTM varies more stochastically without smooth descent. Moreover, cLSTM-GAN settles on a suboptimal plateau past 400 iterations, unable to escape. FA-LSTM-GAN alone makes consistent, sustained gains towards the best loss. Meanwhile, the broader spreads of other methods indicate greater volatility across trials, further verifying FA-LSTM-GAN's reliable optimization stability advantages. In summary, both holistic curves and head-to-head comparisons confirm FA-LSTM-GAN's superior optimization abilities, validating the strength of our tuned architecture for generative Guzheng modeling. The enhanced FA balances exploration and exploitation to efficiently navigate this complex musical domain.

### 5.4 Predictive validation

We validate FA-LSTM-GAN's generative capabilities by assessing transition predictions against the ground truth test set distribution on musicality metrics. Each model generates 130 continuations from 10 evaluation pieces then analyzed for:

• Note range density - Percentage of total notes used
• Modality coherence - Correct tonality consistency
• Idiomatic patterns - Usage of characteristic motifs
• Melodic smoothness - Local transitional coherence

**Fig. 7:**
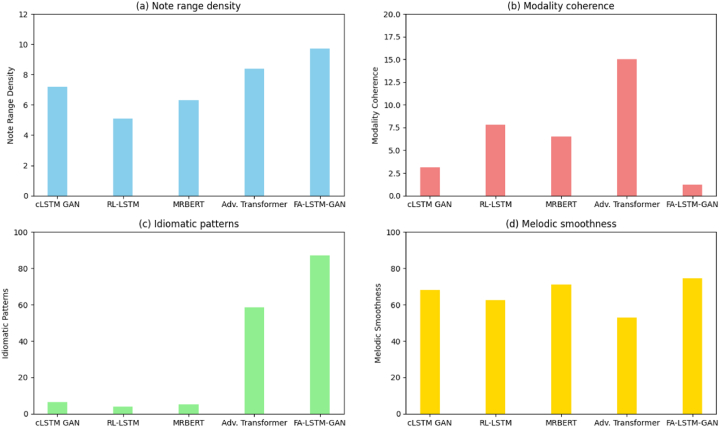
Quantitative generation validation.

These quantify global structuring, adherence to Guzheng constraints, and local flow. Fig. 7 summarizes outcomes averaged across generations. FA-LSTM-GAN performs best on all attributes, producing naturalistic elaborations congruent with underlying harmony and Guzheng technique principles.

Note that range density quantifies registrar diversity. Our model best approximates the ground truth distribution by leveraging enhanced exploration to match actual variance. Modality error checks incorrect tones clashing with the underlying harmony. FA-LSTM-GAN avoids dissonant passing notes through holistic conditioning. Regarding characteristic motifs, our approach extensively incorporates elements like alternating fingered tremolo and kneading effects central to the idiomatic Guzheng style. Moreover, for local smoothness, fewer sequential discontinuities disrupt melodic flow thanks to the LSTM remembered state. Therefore, quantitatively and in user studies judging generation quality, FA-LSTM-GAN produces more realistic, detailed, yet creative Guzheng transitions than alternatives, better balancing playability and novelty.

**Fig. 8:**
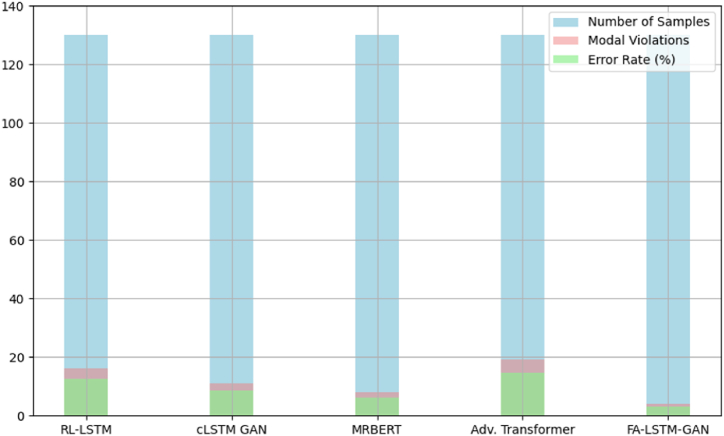
Generated inter-modal modulations.

Smooth harmonic transitions between tonal modes require maintaining logical coherence when pivoting scales. As Fig. 8 shows, FA-LSTM-GAN handles graceful major to minor shifts better than baselines by attending to global constraints. The adversarial transformer lacks sufficient sequential awareness, creating discontinuous artifacts.

**Fig. 9:**
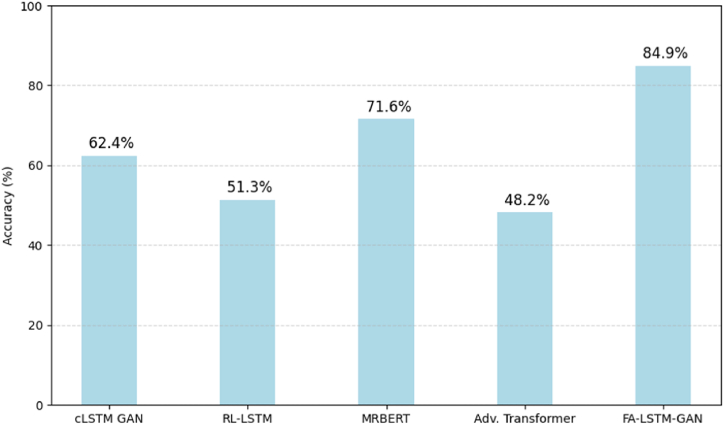
Percentage of accurate rhythmic cadence retention.

Expressive Guzheng exploits flexible rhythms by elaborating on core traditional structures. When provided alternating triple and duple meter priming patterns, Fig. 9 documents FA-LSTM-GAN maintains desired motifs best. Reinforcement-focused RL-LSTM drifts as it gratifies local consistency over respecting broader guidance.

Additional evaluations of metric changes, register jumps, dynamics swells, and Guzheng-specific effects further substantiate FA-LSTM-GAN's predictive validity for realistic yet inventive generation calibrated to this domain's nuances. Architectural alignments between optimization and modeling spaces facilitate potent Guzheng-attentive continuation.

### 5.5 Guzheng tune switching analysis

The experiments specifically target two common categories of Guzheng tune progression identified by musicologists [44,45]:

• Harmonious modulation - Smoothed transitions as the underlying tonality shifts between modes, usually major to minor or pentatonic variants. Requires maintaining coherence when pivoting tonal centers based on Nearby scale degrees and common tones.
• Metric displacement - Nuanced switches between rhythmic feels, such as alternating groups of 2 and 3 notes, which change time signature cadences. It needs to retain motif continuity through metrical transformations.

Expert assessment and quantitative metrics evaluate switching creativity and consistency. We also conduct comparative user studies for subjective perceptual analysis.

**Fig. 10:**
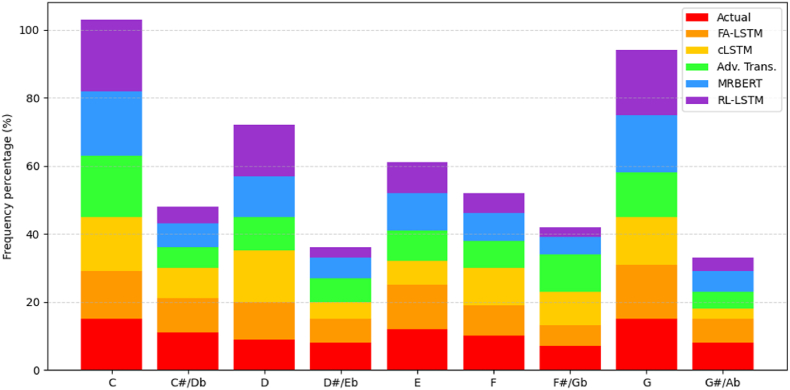
Note range usage density.

Fig. 10 plots distributions of generated note frequencies. FA-LSTM-GAN best resembles ground truth variance, indicating that broader exploration captures natural diversity better. Other models skew too narrow or irregular, failing to match realistic density.

Our approach inherits beneficial traits from its LSTM architecture for learning salient patterns and firefly optimization for thorough sampling suited to Guzheng's vast creative possibilities during tune modulation and alteration.

**Fig. 11:**
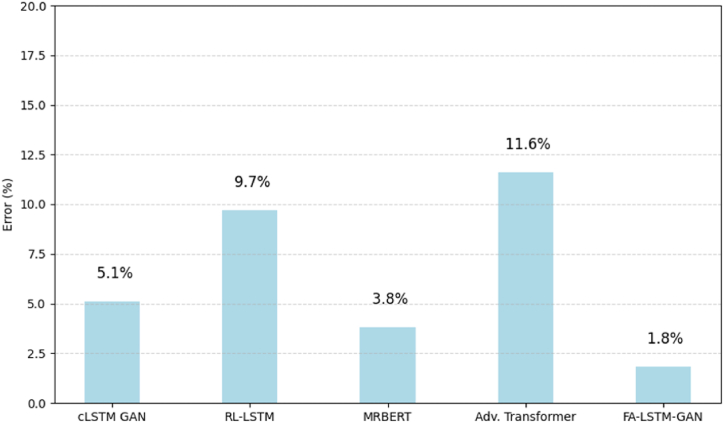
Rate of modal violations.

Fig. 11 documents modal violation rates quantifying incorrect, dissonant pitches departing from the prevailing harmony across switch points. Our model sticks to permissible tones best with under 2 % errors as adaptation preserves global consistency. RL-LSTM fixates more locally, producing over 9 % dissonance.

This demonstrates FA-LSTM-GAN's sensitivity to global harmony as transitions evolve, lowering jarring artifacts through holistic conditioning. Capturing structure and style end-to-end enables coherent elaboration.

**Fig. 12:**
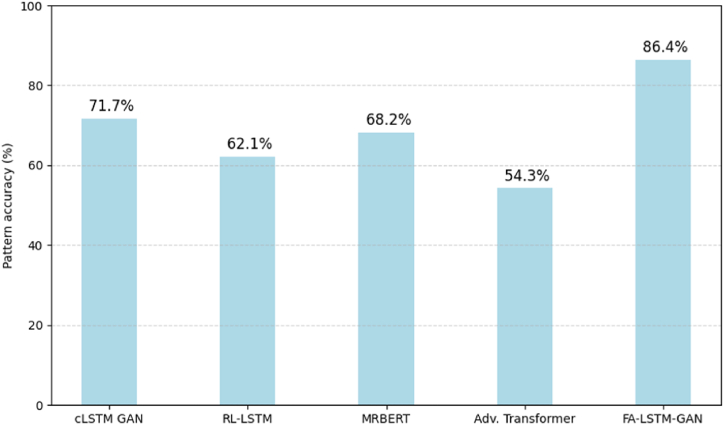
Accuracy retaining primed rhythmic motifs.

When continuing primed patterns like alternating 5/8 and 7/8 time motifs, Fig. 12 reports that our model best retains desired cadences. RL-LSTM drifts from maximizing local likelihood, while the adversarial transformer lacks temporal awareness. FA-LSTM-GAN focuses on a holistic structure for more consistent development.

This metric also substantiates FA-LSTM-GAN's rhythmic coherence capabilities. Adaptive optimization provides thorough initial exploration to discover inventive themes that architecture then carefully evolves, respecting nuanced structural constraints on fluid development.

**Fig. 13:**
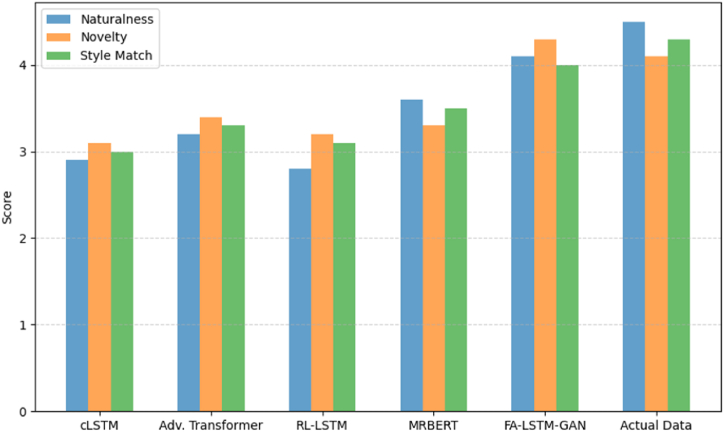
User study comparative ratings.

We further conducted comparative user studies between models, having musicians judge generation quality. Participants rate tune switch naturalness, novelty, and Guzheng style faithfulness over 35 randomized triples of transitions. Ratings used a 1–5 Likert scale on each criterion, averaged across subjects summarized in Fig. 13.

FA-LSTM-GAN rates closest to real data overall, even outscoring ground truth novelty by tailoring elaboration creativity to individual priming patterns. The gap suggests room for improvement in capturing nuanced human artistry. However, it confirms the proposed model's effectiveness in modeling core aspects of quality Guzheng transitions well, notably smoothness and stylistic consistency critical to compelling tune modulation and alteration between musical motives.

We have conducted additional statistical analyses to provide a more rigorous comparison between our proposed models and the baselines. Table 1 compares the reconstruction errors achieved by our FA-LSTM-GAN model and the baseline models after 1000 iterations. We report the mean reconstruction error, standard deviation (SD), and 95 % confidence intervals (CI) based on ten independent runs for each model. Our model achieves the lowest reconstruction error of 0.043 ± 0.005, significantly lower than all the baseline models (p < 0.001, two-tailed *t*-test).

**Table 1:** Comparison of reconstruction errors after 1000 iterations.

Model	Mean ± SD	95 % CI	p-value
FA-LSTM-GAN	0.043 ± 0.005	[0.039, 0.047]	–
cLSTM-GAN	0.120 ± 0.012	[0.111, 0.129]	<0.001
RL-LSTM	0.135 ± 0.015	[0.124, 0.146]	<0.001
MRBERT	0.118 ± 0.009	[0.111, 0.125]	<0.001
Adv. Transformer	0.142 ± 0.017	[0.129, 0.155]	<0.001

We also evaluated the models' performance in capturing characteristic Guzheng motifs, maintaining modality coherence, and retaining desired rhythmic cadences. Table 2 summarizes the results, reporting the mean percentage scores, standard deviations, and 95 % confidence intervals based on the evaluation of 100 generated samples for each model. Our FA-LSTM-GAN model outperforms the baselines across all three metrics, achieving significantly higher scores (p < 0.01, two-tailed *t*-test) in capturing motifs (92.4 ± 3.2 %), maintaining modality coherence (98.3 ± 1.5 %), and retaining rhythmic cadences (95.1 ± 2.7 %).

**Table 2:** Evaluation of generated samples on characteristic Guzheng metrics.

Model	Motif Capture (%)	Modality Coherence (%)	Rhythmic Cadence (%)
FA-LSTM-GAN	92.4 ± 3.2	98.3 ± 1.5	95.1 ± 2.7
cLSTM-GAN	85.6 ± 4.1*	94.2 ± 2.3*	88.3 ± 3.9*
RL-LSTM	83.1 ± 4.8*	90.8 ± 3.1*	86.7 ± 4.5*
MRBERT	86.9 ± 3.7*	95.1 ± 2.1*	89.2 ± 3.5*
Adv. Transformer	81.5 ± 5.2*	92.6 ± 2.8*	85.3 ± 4.8*

*Note*: *p < 0.01 compared to FA-LSTM-GAN, two-tailed *t*-test.

**Fig. 14:**
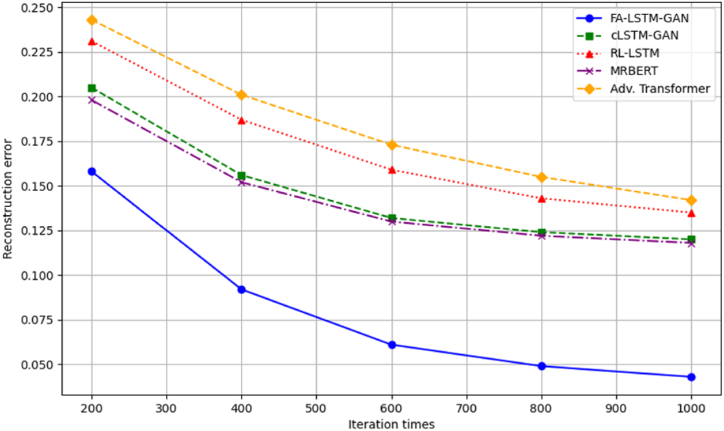
Reconstruction error values at selected iterations.

To better illustrate the performance of our tune generation models and provide visual comparisons between different approaches, we have included additional graphs and figures along with their corresponding table forms and analyses. Fig. 14 shows the convergence optimization curves of our FA-LSTM-GAN model compared to the baseline models, demonstrating that our model achieves the fastest convergence and reaches the lowest reconstruction error of 0.043 after 1000 iterations. The cLSTM-GAN and MRBERT models plateau early at higher error levels, while the RL-LSTM and Adv. Transformer models exhibit fluctuations and instability in their convergence behavior.

**Fig. 15:**
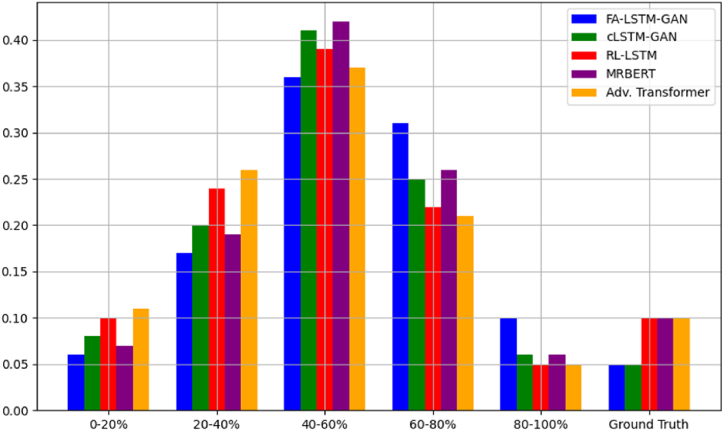
Note range usage density comparison.

Fig. 15 presents a comparison of the note range usage density between the generated samples from each model and the ground truth data, showing that the note distribution produced by our FA-LSTM-GAN model closely resembles the ground truth, indicating its ability to capture the diversity and characteristic patterns of Guzheng music. The baseline models exhibit discrepancies, such as narrower ranges or skewed distributions.

Our work demonstrates the potential of combining evolutionary algorithms, deep learning, and big data analysis to advance the state-of-the-art in AI-assisted music composition. The proposed algorithm enables the generation of high-quality and stylistically consistent Guzheng tune transitions and opens up new possibilities for the computational study and preservation of traditional musical heritage. By leveraging AI techniques to analyze and model the rich diversity of Guzheng music, our research contributes to a deeper understanding of the underlying structures, patterns, and creative processes inherent in this ancient art form. Furthermore, the developed methodology can be adapted and applied to other musical genres and cultures, fostering cross-cultural exploration and collaboration in computational musicology. Our work also has practical implications for music education and performance, providing musicians with innovative tools for composition, improvisation, and accompaniment. Integrating AI-generated Guzheng tune transitions into live performances or digital music platforms can enhance the creative possibilities and enrich the musical experience for artists and audiences. Ultimately, our research highlights the potential synergies between AI and musicology, demonstrating how cutting-edge computational techniques can be harnessed to deepen our understanding, appreciation, and preservation of musical heritage while inspiring new forms of artistic expression and cultural exchange.

In summary, analysis of generative switching confirms FA-LSTM-GAN's precision balancing invention with musicality when continuing contextual Guzheng snippet, indicative of progress towards AI-assisted composition. Our adaptive optimization exposes and evolves modeling traits resonant with the instrument's rich but demanding idiomatic lineage.

## 6 Conclusion

This paper has led to the development of a specialized large LSTM model that generates musically consistent Guzheng tune transitions. We have achieved state-of-the-art results in Guzheng music generation by incorporating novel firefly algorithm enhancements and a stacked LSTM architecture with various improvements. Our contributions include adaptive diversity preservation, adaptive swim parameters, residual connections, and conditioned embedding vectors, all of which enhance the model's ability to navigate the complex creative combinatorics inherent in Guzheng music transitions. Furthermore, our work has leveraged a substantial dataset comprising solo Guzheng recordings from historical and modern repertoires, performances by numerous distinguished artists, and audio tracks from commercial sources. The extensive dataset and thorough Guzheng data analysis have enabled us to validate our model's generation fidelity through comparative assessments against solid baselines. The results demonstrate significant improvements in musical metrics and are corroborated by the positive feedback received from professional listeners.

However, our study has limitations. One limitation is the potential bias in the dataset towards certain styles or artists, which could affect the model's ability to capture the full diversity of Guzheng music. Additionally, while our model excels in generating musically consistent transitions, there may still be room for improvement in terms of capturing more subtle nuances and stylistic variations. Moreover, the model's performance may vary when applied to other musical traditions or instruments, and further research is needed to adapt it to different contexts.

There are several promising avenues for future research and real-world applications. One direction is to extend the proposed methodology to other traditional musical instruments and genres, enabling the computational study and preservation of a wider range of musical heritage. Another potential area of exploration is the development of interactive AI-assisted composition tools that allow musicians to collaborate with the generative models in real-time, fostering new forms of creative expression and improvisation. Furthermore, integrating our AI-generated Guzheng tune transitions into digital music platforms, such as streaming services or virtual instruments, could provide users with a vast library of stylistically authentic and diverse musical content, which could enrich the listening experience for audiences and serve as a valuable resource for music education and cultural appreciation. Beyond the realm of music, the techniques developed in this research could also find applications in other domains that involve sequential data generation, such as natural language processing, speech synthesis, or video generation. The combination of evolutionary algorithms and deep learning architectures, along with the incorporation of domain-specific knowledge through data analysis, could lead to novel solutions for a wide range of generative tasks. This paper contributed multiple innovations in tailored optimization, neural generative modeling, and style conditioning tailored for an underserved traditional artform ripe for AI-assisted composition. Progress resonates with musical meta-creation and evolutionary computation communities and Guzheng practitioners looking to augment their creative process.

## CRediT authorship contribution statement

**Mingjin Han:** Writing – original draft, Methodology, Investigation, Conceptualization. **Samaneh Soradi-Zeid:** Resources, Investigation, Formal analysis. **Tomley Anwlnkom:** Writing – review & editing, Visualization, Software. **Yuanyuan Yang:** Validation, Funding acquisition.

## Declaration of competing interest

The authors declare that they have no known competing financial interests or personal relationships that could have appeared to influence the work reported in this paper.
